# Social Risk Factors That Increase Cardiovascular and Breast Cancer Risk

**DOI:** 10.1007/s11886-023-01957-9

**Published:** 2023-10-06

**Authors:** Omar Obeidat, Kipson R. Charles, Nausheen Akhter, Ann Tong

**Affiliations:** 1 University of Central Florida College of Medicine, Graduate Medical Education/HCA Florida North Florida Hospital, Internal Medicine Residency Program, Gainesville, FL 32605 USA; 2 Division of Cardiology, Department of Medicine, Northwestern University Feinberg School of Medicine, Chicago, IL USA; 3 The Cardiac and Vascular Institute, Gainesville, FL USA

**Keywords:** Cardiovascular disease, Breast cancer, Social risk factors

## Abstract

**Purpose of Review:**

Cardiovascular disease (CVD) and breast cancer (BC) are significant causes of mortality globally, imposing a substantial health burden. This review article aims to examine the shared risk factors and social determinants that contribute to the high prevalence of both diseases, with a focus on social risk factors.

**Recent Findings:**

The common risk factors for CVD and BC, such as hypertension, diabetes, obesity, aging, and physical inactivity, are discussed, emphasizing their modifiability. Adhering to ideal cardiovascular health behaviors has shown a trend toward lower BC incidence. Increased risk of CVD-related mortality is significantly impacted by age and race in BC patients, especially those over 45 years old. Additionally, racial disparities in both diseases highlight the need for targeted interventions. Social determinants of health, including socioeconomic status, education, employment, and neighborhood context, significantly impact outcomes for both CVD and BC.

**Summary:**

Addressing social factors is vital in reducing the burden of both CVD and BC and improving overall health equity.

## Introduction

Cardiovascular disease (CVD) and cancer are two of the leading causes of mortality worldwide, accounting for over one million deaths in the USA alone in 2020 [1, 2]. Breast cancer (BC) continues to be the most common cancer in women, while CVD is the leading cause of death in women, accounting for about one in every five deaths [2]. The risk factors for CVD, including hypertension, diabetes, aging, metabolic syndrome, smoking, and physical inactivity, also contribute to the development of BC [3, 4]. Moreover, BC and its treatments are independent risk factors for CVD, contributing to CVD-related mortality in nearly 16% of women diagnosed with BC [3]. Additionally, CVD may be linked to cancer development [4–7]. This review article highlights the key social factors that play a role in the high prevalence of both CVD and BC. Specifically, we will discuss the impact of socioeconomic status, education, employment, environmental factors, diet, social isolation, physical inactivity, and smoking/alcohol use. It will also explore preventive strategies and future directions to improve both diseases, aiming to reduce the burden and improve health outcomes for at-risk individuals.

## The Burden of CVD and BC

The burden of CVD and BC is a substantial health concern in the USA. In 2020, the prevalence of BC among women in the USA was 3,886,830 cases, with an increasing incidence by 0.5% per year, but declining mortality of 43% through 2020 [8, 9]. In 2021, more than 60 million women have been diagnosed with some form of CVD, and CVD claimed more lives than cancer and chronic lower respiratory disease combined [10]. The number of patients living with BC and CVD has substantially increased as mortality is declining in both diseases over the past 20 years. Survival for BC has increased dramatically, with almost 90% of patients surviving at least 5 years after initial diagnosis [8]. The annual economic burden of CVD and BC in the USA is estimated to be approximately $272.5 billion and $16.5 billion, respectively, and is expected to continue to rise [11–13].

BC survivors face an increased risk of CVD-related mortality compared to women without a history of BC, particularly around 7 years after diagnosis [12]. Therefore, early detection and management of CVD risk factors are essential in mitigating the burden of CVD during this timeframe.

## Common Risk Factors

**Fig. 1:**
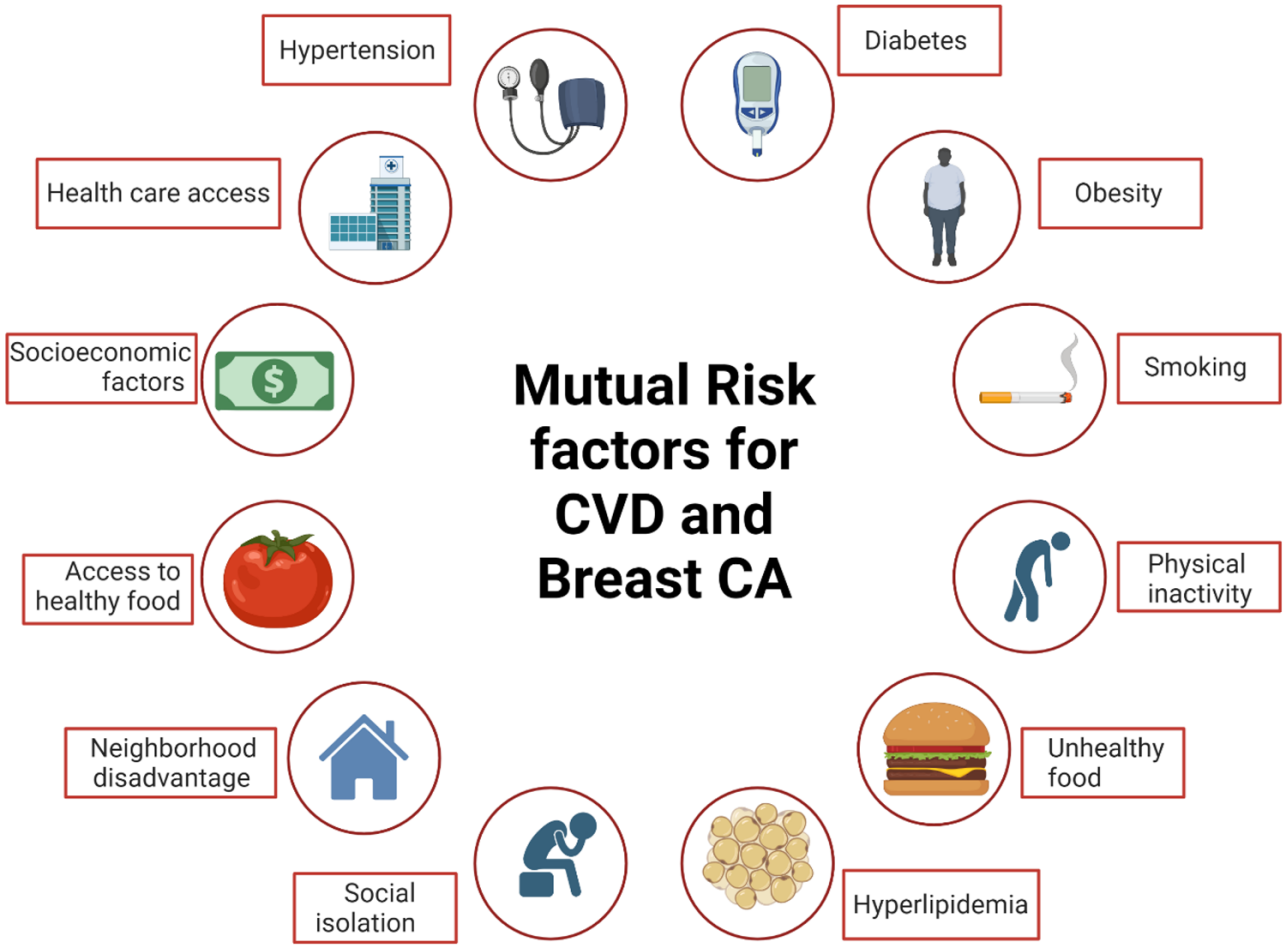
Mutual risk factors of cardiovascular disease and breast cancer. Created with BioRender.com

Growing epidemiological evidence supports the shared risk factors and mechanisms between BC and CVD, aligning with the common soil hypothesis (Fig. 1) [14]. Researchers have emphasized the identification of cardiac risk factors to mitigate the impact of CVD for over six decades. Likewise, BC shares numerous CVD risk factors, including hypertension, obesity, diabetes, and aging, most of which are modifiable [15]. Studies demonstrate that up to 80% of CVD morbidity can be prevented by modifying risk factors through better control of diabetes, dyslipidemia and hypertension, increasing physical activity, consuming a healthy balanced diet, and smoking cessation [16–18]. These risk factors exhibit comparable associations with BC risk, while others demonstrate intricate interactions [15].

Adhering to ideal cardiovascular health behaviors from the American Heart Association’s Life’s Simple 7 has been linked to a trend towards a lower incidence of BC [16]. In a meta-analysis of 18 cohort studies, it was found that women with lower risk profiles have substantially reduced CVD events. Patients with 0, 1, or ≥2 risk factors have an incidence of CVD events of 4.1%, 20.2%, and 30.7%, respectively [15]. Studies have demonstrated that controlling these risk factors is associated with a reduction in lifetime cancer risk [17].

Raising awareness about controlling risk factors has led to increased attention towards preventing BC. Recent discussions have highlighted the importance of identifying and addressing these shared risk factors. Thus, developing effective prevention strategies that can address both BC and CVD requires a better understanding of the common soil hypothesis and the interconnected mechanisms linking both diseases.

## Effect of Age and Race

### Age

The risk of developing BC and CVD increases with age [18, 19]. The lifetime risk of developing BC for women is estimated to be one in eight. Additionally, more than 40% of BC patients are older than 65, and they account for nearly 60% of BC deaths [20]. A study observed that BC patients who did not receive chemotherapy or radiation therapy had a significantly higher standardized mortality ratio (SMR = 2.196, 95% CI: 2.148–2.245, *P* < 0.001) related to CVD compared to the general population [21]. Another study reported SMR for death from heart disease at 10+ years for patients who received radiotherapy and chemotherapy were 2.92 (95% CI 2.81–3.04, *p* < 0.001) and 5.05 (95% CI 4.57–5.55, *p* < 0.001), respectively [22].

### Race

The incidence and mortality rates of CVD and BC are influenced by race and geographic location [23, 24]. Racial disparities exist in the risk of developing BC, with non-Hispanic white women having a higher incidence than other racial groups [23]. Conversely, mortality rates for BC are higher among black females [23]. Black women also have higher incidence and mortality rates for CVD, suggesting a complex relationship between race and disease manifestation [24, 25]. One study reported that the incidence of premenopausal triple negative breast cancer is higher among black females [26]. The etiology of these racial disparities is complex, with multiple social factors contributing to disease susceptibility, including socioeconomic status, lifestyle factors, and barriers to healthcare accessibility [20–26]. A comprehensive understanding of these factors can formulate tailored approaches to reduce the burden of both CVD and BC by race.

## Social Risk Factors

### Social Determinant of Health

Social determinants of health (SDOH), as defined by both the Center for Disease Control (CDC) and the World Health Organization (WHO), encompass a broad range of environmental conditions that impact an individual’s health, well-being, and overall quality of life. These determinants are influenced by various factors, including the places where people live, work, learn, play, worship, and age [27••, 28]. It is widely acknowledged that SDOH has a significant impact on disease outcomes, particularly with respect to CVD and BC [29•, 30].

### Socioeconomic Status

Socioeconomic status (SES) encompasses factors including income, wealth, and education. Racial and ethnic health disparities in the USA are largely driven by financial hardship, housing insecurity, racism, and residential segregation [30]. Higher SES has been linked to a higher incidence of BC compared to women with low SES [31]. This higher incidence is related to differences in risk profiles in women with different SES. Women with high SES have fewer children, have their first child at an older age, and are less likely to breastfeed [31, 32]. Conversely, lower SES is associated with a higher CVD risk [33] and has been linked to worse outcomes in both diseases [33]. Studies reported that BC survival and mortality are associated with the level of education, district of residence, and social class in childhood [34]. BC mortality is significantly higher in blacks compared to non-Hispanic white [35]. Furthermore, a study reported that among adults aged 30 to 59, low SES is associated with a greater than two-fold increase in mortality from ischemic heart disease [36]. Additionally, literature reported that lower income among women was associated with a 10.1-year shorter life span between the poorest and richest individuals [37]. Studies also demonstrated that low SES conditions lead to poor health more frequently than poor health leads to low SES [38]. The difference can be attributed to multiple factors including access to care and higher risk profile [32, 39]. In high-income countries, higher CVD risk factors tend to cluster in low SES populations [40]. The combined effect of individual risk factors is multiplicative rather than additive; therefore, there is a markedly increased risk of CVD and premature disease among individuals of low SES [41]. Furthermore, people with low SES are less likely to receive guideline-recommended medical therapy and cardiac catheterization within 24 hour of acute coronary syndromes [39].

### Education and Employment

Education is needed for health literacy and effective communication with healthcare providers and provides access to higher-paying jobs and stable employment. Literature has demonstrated that a lower educational level is associated with a higher risk of acute MI, CAD, stroke, and heart failure (HF) [42]. It is also associated with a lower incidence of BC [43], however a higher BC mortality rate [44]. Income provides for basic needs such as food, housing, and healthcare services. There is a correlation between income level and the ability to afford a healthier lifestyle and a better-resourced neighborhood, lowering risk of health complications [27••, 29•].

Unemployment is associated with a higher risk of CVD and BC. A French study reported a 20% increase in CAD events among unemployed people without pre-existing heart disease, after adjustment for age, sex, diet, and lifestyle, and nearly 46% of the CAD is explained by dietary and lifestyle factors [45]. A US study reported a 35% increase in acute MI among unemployed people [46]. Even those who are unemployed with high SES have a high risk of CAD events, despite adjustment for covariates such as age, sex, biological characteristics, behavioral variables, and socioeconomic factors. Moreover, the unemployed have worse outcomes in comparison to the retired population [47]. The detrimental effect of unemployment may be driven by the job loss itself or by poor health associated with being unemployed. Employment has a vital impact on health access as health insurance is obtained through employers, and flexibility in the work schedule and paid time off allows individuals to adhere to medical treatment plans [29•, 48]. Black women are more likely to develop BC at a younger age than non-Hispanic white women, increasing the reliance on employer-provided health insurance. However, insufficient sick leave often prevents them from receiving adequate treatment [29•, 49].

Recent research has highlighted the importance of health insurance in promoting health equity, particularly in the context of BC and CVD. Those who are uninsured/underinsured are less likely to have access to preventive medical care and screenings. This can lead to delays in diagnosis and treatment, lower rates of medication adherence and management of chronic illnesses [50]. In BC, advanced stages of the disease have been linked to uninsured black, Indigenous, and Latinx women compared to non-Hispanic whites [51]. Medicaid expansion has been associated with higher mammography screening rates and reduced incidence of advanced BC, with black women and those under 50 years of age achieving the greatest benefit. From 2012 to 2016, there was a significant decrease in the incidence rates of advanced BC in black women in expansion states compared to non-expansion states. In the expansion states, the rates decreased from 24.6 to 21.6%, while in the non-expansion states, the rates remained unchanged at 27.4% [52, 53••].

## Neighborhood Context and Environmental Factors

Neighborhood context plays an important role in health inequities, including disparities in BC and CVD outcomes. Access to resources such as grocery stores, gyms, sports centers, safe places to recreate, and preventive healthcare are essential factors in promoting healthy behaviors [49, 54–56]. In the USA, the impact of the neighborhood is well documented in the literature; zip code can predict life expectancy, health status, and clinical outcomes [57]. Low-income neighborhoods face challenges in healthcare access due to inadequate resources and poor infrastructure. While physical barriers such as housing insecurity, transportation limitations, and travel costs have not been extensively studied [58], high poverty rates and inner-city disadvantages have been associated with worse BC outcomes [59]. Women with non-metastatic BC living in low SES neighborhoods are more likely to present with more aggressive advanced disease and subtypes and have higher disease-specific mortality compared to women in high SES areas [60]. High neighborhood income has been associated with a lower CVD risk. The literature has reported a 10% reduction in acute MI mortality for every $10,000 increase in neighborhood median income [61]. Additionally, neighborhood deprivation, including low income, education, occupation, and housing quality, has been linked to an increased risk of HF and poorer HF outcomes regardless of the SES [62].

Several studies have shown that more individuals in low SES neighborhoods exhibit unhealthy lifestyle behaviors. Specifically, residing in a low SES neighborhood is linked to increased accessibility to fast food establishments [63], reduced engagement in physical exercise, increased sedentary behavior [64], higher likelihood of obesity [65], and tobacco use [66]. These factors, in turn, contribute to various health problems, particularly CVD. Housing instability, including homelessness and difficulty paying rent, is a growing issue that contributes to CVD risk factors and higher CVD mortality rates [67].

In addition to these socioeconomic factors, policy and practice barriers further contribute to health inequities. These policies have led to ongoing racial segregation in the USA, which has significant impacts on health outcomes [49, 54]. Many black patients reside in neighborhoods that limit their ability to achieve optimal health. This can include barriers to obtaining BC and CVD screening and treatment, leading to poorer outcomes for these populations [49, 54]. A study revealed that black women born in Jim Crow states had poorer BC outcomes, including more aggressive forms of cancer, than non-Hispanic white women, regardless of their state of birth [56]. Similarly, the ongoing impact of slavery on heart disease mortality rates has been demonstrated, with black living in regions of the USA with a historical legacy of slavery having worse survival rates from CVD and fewer improvements in disease survival compared to regions that did not practice slavery [55].

Chronic exposure to social and environmental stressors is a major contributor to racial and ethnic inequities in both BC and CVD. Studies have reported that chronic stressors can have a negative impact on health through complex cascades that include the release of epinephrine and norepinephrine. These hormones can result in high blood pressure and poor immune system function, increasing the risk and poor outcomes in both diseases [68]. Epigenetics is a burgeoning field in medicine that focuses on how gene expression is affected by environmental and social factors [69]. Linnenbringer et al. linked this to BC mortality disparities by suggesting that “weathering” of the body’s stress response system may contribute to the expression of BC subtypes with less favorable outcomes [70].

### Diet and Food Insecurity

Diet has been shown to impact the risk of chronic diseases such as CVD and BC. Healthy diets rich in whole grains, fruits, vegetables, and lean proteins have been associated with a lower risk of CVD [71], while unhealthy diets high in saturated fats, added sugars, and sodium have been linked to higher cardiovascular mortality [72]. The effect of diet on BC risk is still controversial, with some studies suggesting a protective effect of a healthy diet, particularly high in fruits and vegetables [73], while others show no significant association [74]. Ultra-processed foods, including sugar-sweetened beverages, have been linked to increased risks of both CVD and BC [75].

There is ongoing research evaluating the influence of dietary factors on BC risk, including the consumption of processed and unprocessed meats. Increased intake of meat has been associated with a higher risk of premenopausal BC, while processed meat intake has shown an association with postmenopausal BC [76]. However, the relationship between diet and BC risk is complex and influenced by various factors such as alcohol consumption and gut microbiota composition. Further investigation is necessary to better understand the impact of diet on BC risk, considering gene-environment interactions and long-term epigenetic mechanisms.

Studies have shown disparities in diet quality based on race, ethnicity, SES, education level, income, and use of food assistance programs in the USA [77–79]. Literature reported that from 1988 to 2010, the percentage of blacks with poor diet was higher than that of non-Hispanic whites by 6.8 to 11.7%, but no difference was observed in 2011 to 2014 due to declining diet quality among non-Hispanic whites [80–83]. Over time, there have been improvements in diet quality among higher-income individuals, while no significant changes were observed for blacks and Mexican Americans [78, 79]. Individuals participating in food assistance programs, such as Supplemental Nutrition Assistance Program (SNAP), generally exhibit suboptimal dietary patterns [81].

Food insecurity (FI) and food deserts are major health problems that contribute to poor dietary patterns and increased risk of CVD. FI refers to limited or uncertain access to adequate food, while food deserts are areas in low-income neighborhoods with limited access to grocery stores providing fresh fruits and vegetables [80]. These challenges make it difficult for individuals to adhere to recommended healthy diets for CVD prevention and management. People experiencing FI and living in food deserts are at greater risk of diet-related CVD, and it is important to recognize and address these issues in clinical settings, particularly among those with existing CVD conditions [81, 82].

### Social Isolation and Loneliness

Social isolation and loneliness are chronic stressors that can have a negative impact on physical and mental health [84]. Changes in society’s structure and demographics have increased the risk of loneliness among individuals [85]. Factors such as increased life expectancy and a growing population of older adults have contributed to reduced social interactions, longer periods of living alone, and a higher prevalence of loneliness among older adults [86]. However, it is important to note that loneliness is not limited to older age but can be encountered at various stages of life [87].

Literature has shown that social networks and connections are important for people with BC and CVD [88]. Adequate social support can have a protective effect on physical and mental health and overall quality of life. Studies have shown that people who lack strong social support have a higher mortality rate, both all-cause mortality and BC mortality. These studies assess social support based on the number of individuals within the social network and the frequency of contact with friends and family following cancer diagnosis [89]. A study that included 2835 participants from the Nurses’ Health Study found that socially isolated participants were twice as likely to die compared to socially connected individuals [89]. Additionally, individuals with robust social support were more likely to adhere to treatment regimens, access healthcare, and effectively utilize treatment options [90].

In a study conducted in 1992, patients with CAD who were unmarried and lacked a confidant experienced a significantly higher 5-year mortality incidence rate compared to those who had a spouse/partner [91]. A recent systematic review and meta-analysis of 16 prospective longitudinal studies revealed a correlation between loneliness and social isolation with an increased risk of CAD (29%) and stroke (32%) [92]. Another cohort study involving 57,825 older women in the USA found that social isolation and loneliness were associated with an 8.0% and 5.0% higher risk of incident CVD, respectively, even after adjusting for health behaviors and outcomes. Women experiencing greater social isolation and loneliness faced a 13 to 27% higher risk of developing CVD compared to those with lower levels of social isolation and loneliness [93].

### Tobacco Use

Tobacco use is a well-established risk factor for CVD [94]. However, its association with BC remains controversial, with conflicting findings reported in the literature. A recent meta-analysis analyzing over 400,000 BC cases found evidence to support a positive dose-response relationship between smoking intensity and BC risk. This may suggest a causal relationship between smoking and BC development [95]. However, other studies have only found weak associations [96]. Additionally, several studies have suggested that the age of smoking initiation is a significant factor in BC risk, with earlier onset associated with a higher risk of developing BC [97]. Furthermore, a meta-analysis of nearly 2.4 million smokers and non-smokers reported that female smokers had a 25% higher risk of developing coronary heart disease than male smokers, with a 95% confidence interval of 1.12 to 1.39 [98].

The effect of passive smoking on BC risk is controversial, with some studies suggesting an increased risk [99] and others finding no significant association [100]. Additionally, some literature suggests that the effect of tobacco use on BC risk is modified by genetic variants in enzymes involved in carcinogen metabolism, particularly NAT2. Patients with slow acetylator variant NAT2 genotypes have a higher risk of developing BC, especially in women with a higher pack year [101].

### Alcohol Use

Alcohol consumption is another well-established risk factor for the development of BC in the scientific literature [102]. Meta-analyses and systematic reviews of epidemiological studies have consistently reported a dose-response relationship between alcohol intake and BC risk. This relationship is most pronounced in patients with estrogen receptor-positive (ER+) BC [3]. In a recent meta-analysis of 22 cohort studies and 45,350 BC cases, the risk of BC increased by 10.5% for every additional 10 g of alcohol consumed per day. This risk was even higher among postmenopausal women, with an increased risk of 11.1% [103]. A review article estimated that in 2012, globally, 144,000 (95% confidence interval [CI]: 88,000 to 200,000) BC cases and 38,000 (95% CI: 2,400 to 53,000) BC deaths were attributable to alcohol consumption, with 18.8% of these cases and 17.5% of these deaths affecting women who were light alcohol consumers [104].

The effect of alcohol consumption on cardiovascular risk is controversial. Observational studies and meta-analyses of observational studies have suggested that light to moderate alcohol intake has a cardioprotective effect on ischemic heart disease [105]. However, heavy alcohol consumption has a deleterious effect on the heart and increased mortality from CVD [105]. Nevertheless, the effect of alcohol consumption on other types of CVD and all-cause mortality remains ambiguous. Recently, Mendelian randomization studies using genetic polymorphisms in enzymes have questioned the beneficial association of low-moderate drinking with the cardiovascular system [106]. As there are considerable variations in the literature, it is difficult to determine a protective effect of moderate alcohol consumption by itself.

## Sedentary Lifestyle and Physical Activity

### Sedentary Lifestyle

Sedentary behavior has been associated with increased risk of CVD and BC [107]. A large observational study involving 71,018 women reported that prolonged sitting more than 10 hours per day was associated with increased CVD risk, even after adjusting for physical activity [108]. Sedentary behavior has also been linked to high breast density, a strong independent risk factor for BC [109]. One case-control study found that sedentary behavior was associated with BC risk, independent of moderate to vigorous activity. Racial differences were observed in the association between sedentary behavior and BC risk, with white women at higher risk than black women [110]. Therefore, reducing sedentary behavior and increasing physical activity are potential targets for CVD and BC prevention intervention.

### Physical Activity

Physical activity has been shown to have benefits for cardiovascular health, including a reduction in the risk of all-cause and cardiovascular mortality, as well as a lower risk of total CVD, coronary heart disease, hypertension, and type 2 diabetes [111]. Only 17.6% of American women meet the recommended guidelines of 150 minutes of moderate-intensity physical activity per week [112]. A growing body of evidence also supports the protective effect of physical activity against BC development among both premenopausal and postmenopausal women, with a linear dose-response relationship observed [113]. A meta-analysis of 29 observational studies found a significant reduction in BC risk among the most physically active women compared to the least active [114]. Furthermore, a more recent meta-analysis of 22 studies involving 123,574 participants found an inverse relationship between physical activity and BC events and deaths. Women who engage in high levels of lifetime recreational physical activity have a significantly lower risk of BC-related death compared to those who report low or no recreational physical activity, and physical activity during adolescence may also lower the risk of premenopausal BC [115]. Epidemiological studies have primarily focused on the benefits of aerobic activities, and more research is needed to explore the role of muscle-strengthening activities in cancer prevention [116]. The underlying biological mechanisms that explain the protective effect of physical activity against BC remain unclear, although studies suggest potential hormonal and non-hormonal pathways. Hormonal pathways may involve reduced levels of estrogen in postmenopausal women, while non-hormonal pathways may involve immune function, inflammation, oxidative stress, myokines, insulin, insulin-like growth factors, and adipokines [117].

## Future Directions

There is an increasing recognition of social risk factors’ significance in CVD and BC outcomes, leading to the development of targeted interventions to address health disparities. However, more work remains to be done. Improving access to healthcare and addressing social determinants of health are key areas that require attention. This involves enhancing affordable and comprehensive healthcare access, promoting healthy behaviors through environmental improvements, and addressing social and economic inequalities contributing to health disparities. Interventions such as the SNAP can mitigate food insecurity, while collaboration among healthcare providers, policymakers, community partners, and patients is crucial for developing comprehensive intervention strategies. By improving education and healthcare access as well as addressing housing, food, and transportation issues, policy makers can help reduce inequality. Addressing other issues including racial segregation, institutional racism, and social isolation are all important components of this comprehensive approach (Table 1).

**Table 1:** The mitigation strategies for social risk factors in cardiovascular disease and breast cancer

**Domain**	**Strategy**	**Possible solutions**
**Income stability**	Expand health insurance coverage	Patient Protection and Affordable Care Act (ACA), Medicaid expansion, Children’s Health Insurance Program (CHIP)
	Create a robust income safety net	Provide financial assistance to low-income individuals and families
	Increase income benefits	Expand access to social programs, such as food stamps and housing assistance
	Increase jobs/employment	Provide job training and placement services to help people find work
		Raise the minimum wage
		Provide tax breaks for low-income workers
		Invest in infrastructure and education.
		Provide tax breaks for businesses that create jobs
		Make it easier for people to start their own businesses
**Education access and quality**	Achieve health literacy within healthcare organizations	Provide education to hospital staff and patients, collaborate with local communities to develop programs centered on increasing health literacy or high school graduation rates
**Healthcare access and quality**	Improve primary care access	Focus on patient-centered medical homes, improve access to specialists
**Housing**	Address structural inequities	Provide financial support to organizations that house homeless people, develop or partner with medical respite programs
**Tobacco control**	Well-coordinated interventions	Identify tobacco users at each healthcare encounter, advocate for increased coverage of tobacco-dependence treatments, participate in programs that provide financial incentives for tobacco cessation
**Physical activity**	Support multidisciplinary teams	Exercise physiologists, nutritionists, and social workers working together to develop personalized fitness programs for individuals with cardiometabolic risk factors
**Racial segregation**	Increase understanding of community needs and barriers to equitable care	Partner with nontraditional treatment locations such as barbershops and faith-based centers, utilizing e-prescribing systems like CommunityRx to connect patients to health-promoting community resources
**Sex and gender equity**	Implement policies and programs to improve equity in care	Establish dedicated women’s cardiovascular prevention clinics, provide curricula on understanding the needs of transgender patients, increase workforce and trainee diversity
**Social isolation**	Implement a systematic approach to identify individuals at high risk and provide resources for management	Expand telehealth resources and utilize social media targeted towards local communities
**Food and nutrition insecurity**	Actively enroll eligible patients in federal and local food programs	Develop programs and collaborate with community resources such as food pantries, double-up food bucks participants, and produce prescriptions, in-clinic food pantries and participation in state programs that integrate “food is medicine” concepts into healthcare, such as medically tailored meals

Furthermore, increasing awareness and education on social risk factors for CVD and BC among healthcare providers and patients is crucial, along with the development of culturally sensitive interventions. Public awareness and education efforts can help reduce stigma and improve health behaviors. Additionally, further research is needed to understand the mechanisms through which social risk factors influence disease. This entails identifying biomarkers and molecular pathways affected by social factors and creating targeted interventions. Identifying vulnerable subgroups, such as those with specific genetic and physiological profiles, is also essential in advancing understanding and intervention strategies related to social risk factors. By implementing these mitigation strategies, it is possible to improve health outcomes and reduce disparities associated with CVD and BC.

## Conclusion

CVD and BC are multifaceted conditions influenced by various clinical, social, and environmental factors. Despite the significance of genetic and physiological components in the development and progression of these diseases, social determinants of health such as socioeconomic status, education level, race/ethnicity, environmental exposures, and psychosocial factors also have a substantial impact on disease risk and outcomes. To mitigate health disparities and devise effective interventions, it is crucial to comprehend the social risk factors involved in the etiology of CVD and BC and their underlying mechanisms. Addressing social risk factors can lead to a future where all individuals receive equitable prevention and treatment for these diseases, irrespective of their social or economic status.

## Abbreviations

CVD: Cardiovascular disease
BC: Breast cancer
SMR: Standardized mortality ratio
SES: Socioeconomic status
MI: Myocardial infarction
CAD: Coronary artery disease
HF: Heart failure
SNAP: Supplemental Nutrition Assistance Program
AA: African American
WHO: World Health Organization
CDC: Centers for Disease Control and Prevention
